# Organizational and Psychological Determinants of Nursing Performance: A PLS-SEM Analysis Among Registered Nurses in Saudi Arabia

**DOI:** 10.12688/f1000research.186749.1

**Published:** 2026-09-11

**Authors:** Adawi Dalal Othman, Hafizah Che Hassan

**Affiliations:** 1 Lincoln University College, Petaling Jaya, Selangor, Malaysia

**Keywords:** work-life balance, nursing performance, employee well-being, stress reduction, nurses, PLS-SEM, occupational health, Saudi Arabia.

## Abstract

**Background:**

Maintaining high nursing performance is increasingly challenging in modern healthcare systems due to heavy workloads, workforce shortages, and growing psychological demands. Although work-life balance has been recognized as an important organizational resource, limited evidence has examined how it influences nursing performance through employee well-being and stress reduction, particularly in Saudi Arabian healthcare settings. This study investigated the relationships among work-life balance, employee well-being, stress reduction, and nursing performance among registered nurses.

**Methods:**

A cross-sectional study was conducted among 427 registered nurses employed at The Medical City of Medina, Saudi Arabia. Participants were selected using proportionate stratified random sampling. Data were collected using validated self-administered questionnaires and analysed using Partial Least Squares Structural Equation Modelling (PLS-SEM). Direct and indirect relationships were evaluated using a bootstrapping procedure with 5,000 resamples.

**Results:**

Work-life balance demonstrated significant positive effects on employee well-being (β = 0.885, *p* < 0.001), stress reduction (β = 0.888, *p* < 0.001), and nursing performance (β = 0.219, *p* < 0.001). Stress reduction emerged as the strongest predictor of nursing performance (β = 0.417, *p* < 0.001), followed by employee well-being (β = 0.320, *p* < 0.001). Both employee well-being (β = 0.283, *p* < 0.001) and stress reduction (β = 0.371, *p* < 0.001) significantly mediated the relationship between work-life balance and nursing performance.

**Conclusions:**

Work-life balance contributes to nursing performance both directly and indirectly by improving employee well-being and reducing occupational stress. These findings highlight the importance of organizational strategies that promote healthy work environments, flexible work arrangements, and psychological support programmes to strengthen nursing performance and workforce sustainability in tertiary healthcare settings.

## Introduction

Nurses constitute the largest professional workforce within healthcare systems and play a fundamental role in ensuring patient safety, healthcare quality, and continuity of care. The quality of nursing performance has become increasingly important as healthcare organizations worldwide continue to experience growing service demands, workforce shortages, increasing administrative responsibilities, and the long-term consequences of the COVID-19 pandemic. These challenges have intensified nurses’ workloads, increased occupational stress, and highlighted the importance of developing sustainable workforce strategies that promote both employee well-being and high-quality patient care ( World Health Organization, 2020; Dall'Ora et al., 2020; Zhang et al., 2024).

Among the organizational factors influencing workforce sustainability, work-life balance (WLB) has received increasing attention in nursing research. Work-life balance refers to an individual’s ability to effectively balance professional responsibilities with personal and family life. In nursing practice, maintaining an appropriate work-life balance is particularly challenging because nurses frequently work rotating shifts, extended working hours, and emotionally demanding clinical environments. Previous studies have consistently demonstrated that inadequate work-life balance is associated with burnout, emotional exhaustion, reduced job satisfaction, increased turnover intention, and poorer quality of patient care. Conversely, supportive organizational policies, flexible work arrangements, and healthy work environments have been associated with greater employee engagement, improved workforce retention, and enhanced organizational effectiveness ( Fukuzaki et al., 2021; Helaly, 2022; Zhang et al., 2024; Ariasari & Tjahjono, 2024).

Although evidence consistently supports a positive association between work-life balance and employee outcomes, the processes through which work-life balance contributes to nursing performance remain incompletely understood. The Job Demands–Resources (JD-R) theory proposes that organizational resources enable employees to cope more effectively with demanding work environments, thereby improving motivation, engagement, and job performance ( Bakker & Demerouti, 2007). Similarly, the Conservation of Resources (COR) theory suggests that individuals seek to acquire, maintain, and protect valuable personal and occupational resources, whereas resource loss contributes to stress, diminished well-being, and impaired work performance ( Hobfoll, 1989). Within these theoretical perspectives, employee well-being and stress reduction may represent important organizational and psychological factors through which work-life balance is associated with nursing performance.

Despite the growing body of literature on work-life balance among healthcare professionals, several important knowledge gaps remain. Existing studies have primarily focused on direct associations between work-life balance and outcomes such as burnout, job satisfaction, and psychological well-being, whereas comparatively fewer studies have explored the combined roles of employee well-being and stress reduction in relation to nursing performance. Furthermore, most available evidence has been generated in Western countries and East Asia, with relatively limited research conducted in Middle Eastern healthcare systems despite differences in organizational structures, workforce composition, and sociocultural contexts ( Alfuqaha et al., 2021). Evidence from Saudi Arabian tertiary hospitals is particularly scarce, limiting the applicability of current knowledge to healthcare organizations operating within this setting.

Addressing these knowledge gaps is especially important in Saudi Arabia, where continuing healthcare reforms, increasing service demands, and the need to sustain a resilient nursing workforce require organizational strategies that effectively support nurses’ well-being and professional performance. A clearer understanding of the relationships among work-life balance, employee well-being, stress reduction, and nursing performance may provide healthcare administrators with evidence to design organizational interventions that strengthen workforce sustainability while maintaining high standards of patient care.

Therefore, this study aimed to examine the relationships among work-life balance, employee well-being, stress reduction, and nursing performance among registered nurses at The Medical City of Medina, Saudi Arabia. Guided by the Job Demands–Resources (JD-R) theory and the Conservation of Resources (COR) theory, the study sought to provide context-specific evidence regarding organizational and psychological factors associated with nursing performance in Saudi Arabian healthcare settings.

Specifically, the objectives of this study were: (1) To examine the direct effect of work-life balance on nursing performance among registered nurses; (2) To evaluate the effects of work-life balance on employee well-being and stress reduction; (3) To examine the effects of employee well-being and stress reduction on nursing performance; (4) To investigate the mediating roles of employee well-being and stress reduction in the relationship between work-life balance and nursing performance.

The findings are expected to provide empirical evidence to inform organizational policies aimed at improving nurses’ well-being, reducing occupational stress, strengthening workforce sustainability, and enhancing the quality of healthcare delivery.

## Methods

### Study design and setting

This analytical cross-sectional study was conducted among registered nurses at The Medical City of Medina, Saudi Arabia, to examine the relationship between work-life balance and nursing performance and to investigate the potential mediating roles of employee well-being and stress reduction. The study was reported in accordance with the Strengthening the Reporting of Observational Studies in Epidemiology (STROBE) Statement for cross-sectional studies ( von Elm et al., 2007).

Data were collected from nurses working in major clinical departments, including medical, surgical, emergency, intensive care, and outpatient services. Because the study aimed to examine associations among organizational and psychological factors at a single point in time, a cross-sectional design was considered appropriate.

### Participants and sampling

The target population comprised 520 registered nurses employed at The Medical City of Medina. Eligible participants were registered nurses who: were employed full-time; had at least six months of clinical experience at the hospital; and provided written informed consent before participation. Nurses on extended leave during the study period and personnel without direct patient-care responsibilities were excluded.

The minimum required sample size was determined using the Krejcie and Morgan (1970) sample size table for finite populations at a 95% confidence level and a 5% margin of error, indicating that at least 222 participants were required. To maximize statistical power and reduce the potential effect of non-response, proportionate stratified random sampling was performed across clinical departments, and all eligible nurses within each stratum were invited to participate. A total of 427 completed questionnaires were included in the final analysis, corresponding to a response rate of 82.1%.

### Study instrument

Data were collected using a structured self-administered questionnaire comprising five sections. The first section collected participants’ demographic characteristics, including age, sex, marital status, educational level, years of nursing experience, and work schedule.

The remaining sections assessed four latent constructs: work-life balance (8 items), employee well-being (10 items), stress reduction (9 items), nursing performance (7 items). The work-life balance scale was adapted from Fisher-McAuley et al. (2003) and Haar et al. (2014). Employee well-being items were adapted from Clark (2000) and Robertson and Cooper (2010). Stress reduction items were developed based on Maslach and Leiter (2016) and Helaly (2022), while nursing performance items were adapted from Laschinger et al. (2014) and Zhang et al. (2024). All questionnaire items were rated using a five-point Likert scale ranging from 1 (“strongly disagree”) to 5 (“strongly agree”), with higher scores indicating higher levels of the respective construct.

### Pilot testing

Prior to the main survey, the questionnaire was pilot tested among 20 registered nurses who were not included in the final study sample. The pilot study assessed questionnaire clarity, comprehensibility, and preliminary reliability. Internal consistency was evaluated using Cronbach’s alpha coefficients. All constructs demonstrated acceptable reliability, with alpha values exceeding the recommended threshold of 0.70. Cronbach’s alpha coefficients were 0.87 for work-life balance, 0.90 for employee well-being, 0.88 for stress reduction, and 0.86 for nursing performance, indicating satisfactory internal consistency of the measurement instrument.

### Data collection

Data were collected using both paper-based and electronic questionnaires. Departmental nurse managers assisted in distributing study invitations during departmental meetings and shift handovers. Before participation, eligible nurses received written information describing the study objectives, voluntary nature of participation, confidentiality safeguards, and their right to withdraw at any time without penalty. Questionnaires were completed anonymously. Responses were screened for completeness before analysis, and questionnaires containing substantial missing data were excluded.

### Statistical analysis

Data were analysed using SmartPLS version 4 (SmartPLS GmbH, Oststeinbek, Germany). Partial Least Squares Structural Equation Modelling (PLS-SEM) was employed to examine the hypothesized relationships among work-life balance, employee well-being, stress reduction, and nursing performance because it enables the simultaneous estimation of complex relationships among latent variables while accommodating mediation analysis ( Hair et al., 2022).

Descriptive statistics were first calculated to summarize participants’ demographic characteristics and study variables. Pearson correlation analysis was performed to examine bivariate relationships among the constructs before structural model evaluation.

The PLS-SEM analysis followed the two-step approach recommended by Hair et al. (2022). First, the measurement model was evaluated by assessing indicator reliability, internal consistency reliability using Cronbach’s alpha and Composite Reliability (CR), convergent validity using the Average Variance Extracted (AVE), and discriminant validity using the Heterotrait–Monotrait ratio (HTMT). Variance Inflation Factors (VIFs) were also examined to assess potential multicollinearity among predictor constructs.

Second, the structural model was evaluated by estimating standardized path coefficients (β), coefficients of determination (R ^2^), effect sizes (f ^2^), predictive relevance (Q ^2^), and the standardized root mean square residual (SRMR). The significance of direct and indirect effects was assessed using a non-parametric bootstrapping procedure with 5,000 resamples. Statistical significance was established at p < 0.05.

## Results

### Participant characteristics

A total of 427 registered nurses participated in this study, corresponding to a response rate of 82.1%. Most participants were female, aged between 25 and 44 years, held a bachelor’s degree, and had between five and ten years of professional nursing experience ( Table 1).

**Table 1.** Demographic profile of respondents.

Demographic Variable	Category	Frequency (n)	Percentage (%)
**Gender**	Female	320	74.9%
	Male	107	25.1%
**Age**	Under 25	43	10.1%
	25–34	171	40.0%
	35–44	128	30.0%
	45–54	64	15.0%
	55+	21	4.9%
**Marital Status**	Single	128	30.0%
	Married	256	60.0%
	Divorced	34	8.0%
	Widowed	9	2.0%
**Education**	Diploma	85	19.9%
	Bachelor	278	65.1%
	Master	55	12.9%
	Doctorate	9	2.1%
**Experience**	< 5 years	85	19.9%
	5–10 years	171	40.0%
	11–15 years	107	25.1%
	> 15 years	64	15.0%
**Work Schedule**	Day	171	40.0%
	Night	85	19.9%
	Rotational	171	40.0%

### Measurement model assessment

The measurement model demonstrated satisfactory reliability and convergent validity ( Table 2). Cronbach’s alpha values ranged from 0.876 to 0.893, while composite reliability values ranged from 0.902 to 0.914, exceeding the recommended threshold of 0.70. Average Variance Extracted (AVE) values ranged from 0.535 to 0.572, confirming adequate convergent validity. In addition, all indicator outer loadings exceeded the recommended threshold of 0.70, indicating satisfactory indicator reliability.

**Table 2.** Measurement Model Assessment.

Construct	Cronbach’s α	Composite Reliability	AVE
Work-Life Balance	0.879	0.904	0.542
Employee Well-Being	0.876	0.902	0.535
Stress Reduction	0.893	0.914	0.572
Nursing Performance	0.882	0.906	0.548

*Note*: Cronbach’s α = Cronbach’s alpha; AVE = Average Variance Extracted. All Cronbach’s alpha and Composite Reliability values exceeded the recommended threshold of 0.70, while all AVE values exceeded 0.50, indicating satisfactory internal consistency reliability and convergent validity ( Hair et al., 2022).

### Structural model assessment

The structural model demonstrated substantial explanatory power ( Table 3). Work-life balance explained 78.3% of the variance in employee well-being (R ^2^ = 0.783) and 78.9% of the variance in stress reduction (R ^2^ = 0.789). Collectively, work-life balance, employee well-being, and stress reduction explained 84.7% of the variance in nursing performance (R ^2^ = 0.847). Furthermore, the standardized root mean square residual (SRMR = 0.044) indicated satisfactory overall model fit.

**Table 3.** Structural Model Results.

Endogenous Construct	R ^2^
Employee Well-Being	0.783
Stress Reduction	0.789
Nursing Performance	0.847

*Note*: SRMR = Standardized Root Mean Square Residual. An SRMR value below 0.08 indicates acceptable model fit.

### Hypothesis testing

The results of the bootstrapping analysis supported all proposed hypotheses ( Table 4). Work-life balance exerted significant positive effects on employee well-being (β = 0.885, *t* = 87.659, *p* < 0.001), stress reduction (β = 0.888, *t* = 94.286, *p* < 0.001), and nursing performance (β = 0.219, *t* = 4.653, *p* < 0.001). Employee well-being also showed a significant positive association with nursing performance (β = 0.320, *t* = 6.878, *p* < 0.001), while stress reduction emerged as the strongest direct predictor of nursing performance (β = 0.417, *t* = 8.990, *p* < 0.001).

**Table 4.** Direct Effects (Hypothesis Testing).

Hypothesis	Path	β	t-value	p-value	Decision
H1	Work-Life Balance → Employee Well-Being	0.885	87.659	<0.001	Supported
H2	Work-Life Balance → Stress Reduction	0.888	94.286	<0.001	Supported
H3	Work-Life Balance → Nursing Performance	0.219	4.653	<0.001	Supported
H4	Employee Well-Being → Nursing Performance	0.320	6.878	<0.001	Supported
H5	Stress Reduction → Nursing Performance	0.417	8.990	<0.001	Supported

### Mediation analysis

The mediation analysis demonstrated that both employee well-being and stress reduction significantly mediated the relationship between work-life balance and nursing performance ( Table 5). The indirect effect of work-life balance on nursing performance through employee well-being was statistically significant (β = 0.283, *t* = 6.904, *p* < 0.001). Likewise, stress reduction significantly mediated this relationship (β = 0.371, *t* = 8.918, *p* < 0.001). Because both the direct and indirect effects remained statistically significant, the findings indicate partial mediation.

**Table 5.** Mediation Analysis.

Indirect Path	β	t-value	p-value	Interpretation
Work-Life Balance → Employee Well-Being → Nursing Performance	0.283	6.904	<0.001	Significant partial mediation
Work-Life Balance → Stress Reduction → Nursing Performance	0.371	8.918	<0.001	Significant partial mediation

## Discussion

The present study examined the relationships among work-life balance, employee well-being, stress reduction, and nursing performance among registered nurses working in a tertiary healthcare institution in Saudi Arabia using Partial Least Squares Structural Equation Modelling (PLS-SEM). Overall, the findings supported the proposed conceptual framework and demonstrated that work-life balance influenced nursing performance both directly and indirectly through employee well-being and stress reduction. Notably, stress reduction emerged as the strongest predictor of nursing performance, followed by employee well-being, indicating that the beneficial effects of work-life balance are largely transmitted through psychological mechanisms that enable nurses to cope with demanding clinical environments.

The significant direct association between work-life balance and nursing performance is consistent with a growing body of evidence indicating that nurses who successfully balance professional and personal responsibilities are more likely to maintain higher levels of work engagement, productivity, and quality of care. In modern healthcare systems, nurses routinely experience heavy workloads, rotating shifts, emotional labour, and increasing administrative responsibilities. These occupational demands may impair cognitive functioning, increase fatigue, and reduce clinical performance when opportunities for recovery are insufficient. Conversely, supportive work environments that facilitate work-life balance promote resilience, professional commitment, and sustained job performance, thereby contributing to better patient care and workforce retention ( Fukuzaki et al., 2021; Zhang et al., 2024).

A major contribution of the present study is the finding that stress reduction was the strongest direct predictor of nursing performance. Although previous studies have frequently emphasized employee well-being as the principal psychological determinant of work performance, the current findings suggest that minimizing occupational stress may have an even greater influence on nurses’ ability to perform effectively in high-demand clinical settings. Persistent occupational stress has been associated with impaired concentration, emotional exhaustion, reduced decision-making capacity, and increased risk of clinical errors. Therefore, organizational initiatives aimed at reducing workplace stress may produce substantial improvements in nursing performance and patient safety. Recent studies have likewise reported that stress management, supportive leadership, and healthy working environments are strongly associated with improved workforce outcomes and organizational effectiveness ( Stankevičienė et al., 2021; Zhang et al., 2024).

Employee well-being also demonstrated a significant positive association with nursing performance and acted as an important mediator between work-life balance and professional performance. This finding reinforces previous evidence suggesting that psychologically healthy nurses are more engaged, resilient, and capable of maintaining high-quality patient care despite demanding workplace conditions ( Dousin et al., 2019; Haider et al., 2018). Employee well-being encompasses positive emotional functioning, psychological resilience, and life satisfaction, all of which facilitate sustained professional performance. The significant indirect pathway identified in this study indicates that organizational strategies designed to improve work-life balance may enhance nursing performance partly by strengthening nurses’ psychological well-being.

The mediation analysis provides further insight into the mechanisms linking work-life balance with nursing performance. Both employee well-being and stress reduction significantly mediated this relationship, indicating that work-life balance contributes to improved performance not only through its direct influence but also through its capacity to preserve psychological resources and reduce occupational strain. Because both the direct and indirect effects remained statistically significant, the findings support a partial mediation model, suggesting that additional organizational and individual factors may also contribute to nursing performance.

These findings provide empirical support for the Job Demands-Resources theory ( Bakker & Demerouti, 2007). According to the JD-R framework, job resources buffer the adverse effects of high job demands while promoting motivation and performance. Within the present study, work-life balance can be interpreted as an important organizational resource that enables nurses to recover from demanding clinical work, thereby enhancing employee well-being, reducing stress, and ultimately improving nursing performance. The substantial explanatory power of the structural model further supports the proposition that organizational resources play a central role in sustaining workforce performance in healthcare settings.

The findings are also consistent with the Conservation of Resources theory ( Hobfoll, 1989), which proposes that individuals seek to acquire, preserve, and protect valuable personal resources. When nurses experience prolonged work-life conflict, these resources become depleted, leading to psychological distress and reduced occupational functioning. Conversely, organizational practices that support work-life balance help preserve emotional and cognitive resources, allowing nurses to maintain better psychological well-being and lower stress levels. The simultaneous application of the JD-R and COR theories therefore provides a comprehensive theoretical explanation for the observed direct and indirect relationships identified in this study.

An additional strength of this study lies in its contribution to the relatively limited literature from the Middle East. Most previous investigations examining work-life balance and nursing outcomes have been conducted in Europe, North America, and East Asia, whereas evidence from Saudi Arabia remains comparatively scarce. Differences in healthcare systems, workforce composition, organizational culture, and sociocultural expectations may influence how nurses experience work-life balance and occupational stress. Consequently, the present findings provide context-specific evidence that extends the applicability of the JD-R and COR theories within Saudi Arabian tertiary healthcare institutions and contributes to the growing international literature on nursing workforce sustainability.

From a practical perspective, the findings suggest that healthcare organizations should move beyond traditional productivity-focused management approaches and adopt comprehensive workforce strategies that simultaneously strengthen work-life balance, promote employee well-being, and reduce occupational stress. Interventions such as flexible work scheduling, adequate staffing, supportive leadership, employee assistance programmes, psychological counselling services, and structured workplace wellness initiatives may enhance nurses’ psychological resilience while improving workforce retention, patient safety, and healthcare quality. These findings reinforce the view that investments in nurses’ well-being represent strategic investments in organizational performance rather than solely employee welfare initiatives.

### Strengths and limitations

This study has several strengths. First, it included a relatively large sample of registered nurses recruited from multiple clinical departments within a tertiary healthcare institution. Second, the study was guided by two complementary theoretical frameworks, the JD-R and COR theories,which enabled a comprehensive examination of both organizational and psychological mechanisms underlying nursing performance. Third, the application of PLS-SEM allowed simultaneous estimation of direct and indirect relationships, providing a more comprehensive understanding of the mediating roles of employee well-being and stress reduction.

Nevertheless, several limitations should be acknowledged. The cross-sectional design precludes causal inference, and the use of self-administered questionnaires may have introduced common method and social desirability bias. In addition, data were collected from a single tertiary hospital in Saudi Arabia, which may limit the generalizability of the findings to other healthcare settings. Finally, although the proposed model explained a substantial proportion of the variance in nursing performance, other potentially relevant determinants, including leadership style, organizational culture, psychological safety, staffing adequacy, resilience, burnout, and work engagement, were not examined and warrant further investigation.

### Future research

Future research should employ longitudinal or prospective study designs to establish causal relationships among work-life balance, employee well-being, stress reduction, and nursing performance. Multi-centre studies involving different healthcare organizations and countries would enhance the external validity of the findings. Further studies should also incorporate additional organizational and psychological constructs, including transformational leadership, organizational support, burnout, resilience, psychological safety, and employee engagement, to develop a more comprehensive understanding of workforce performance. Intervention studies evaluating organizational programmes designed to improve work-life balance and reduce occupational stress would provide stronger evidence to inform nursing management and healthcare workforce policies.

## Conclusion

This study demonstrates that work-life balance is an important organizational resource associated with improved nursing performance among registered nurses working in a tertiary healthcare institution in Saudi Arabia. Using Partial Least Squares Structural Equation Modelling (PLS-SEM), the findings indicate that work-life balance exerts both direct and indirect effects on nursing performance through employee well-being and stress reduction. Among the examined predictors, **stress reduction emerged as the strongest determinant of nursing performance**, followed by employee well-being, highlighting the critical role of psychological health in supporting nurses’ professional performance.

The significant mediating effects of employee well-being and stress reduction suggest that improving work-life balance alone may be insufficient unless accompanied by organizational strategies that promote psychological well-being and minimize occupational stress. These findings support the theoretical propositions of the Job Demands–Resources (JD-R) and Conservation of Resources (COR) theories, emphasizing that organizational resources contribute to improved workforce performance by preserving employees’ psychological and emotional resources.

From a practical perspective, healthcare organizations should implement comprehensive workforce policies that integrate flexible work arrangements, adequate staffing, supportive leadership, workplace wellness programmes, and accessible psychological support services. Such organizational interventions may strengthen nurses’ well-being, reduce occupational stress, improve workforce sustainability, and ultimately enhance the quality and safety of patient care.

Although this study provides important evidence from a Saudi Arabian tertiary healthcare setting, future longitudinal and multi-centre studies are warranted to confirm the causal relationships identified and to examine additional organizational and psychological determinants of nursing performance across diverse healthcare contexts.

### Ethical considerations

This study was conducted in accordance with the ethical principles of the Declaration of Helsinki. Ethical approval was obtained from the Research Ethics Committee of Lincoln University College, prior to the commencement of the study (Approval No. LUC/MKT/SP/007/223). Written informed consent was obtained from all participants before data collection. Participants were informed about the purpose of the study, the voluntary nature of participation, their right to withdraw at any time without penalty, and the confidentiality of their responses. All data were de-identified prior to analysis and used solely for research purposes.

## Data Availability

The de-identified dataset supporting the findings of this study is available from Zenodo: Adawi Dalal, O., & Hafizah Che, H. (2026). *Data Sheet: Work-Life Balance and Nursing Performance Among Registered Nurses in Saudi Arabia* [Dataset]. Zenodo. https://doi.org/10.5281/zenodo.21293834. The dataset contains anonymized questionnaire responses and does not include personally identifiable information. The study materials supporting this research are available from Zenodo: Adawi Dalal, O., & Hafizah Che, H. (2026). *Extended Data for: Organizational and Psychological Determinants of Nursing Performance: A PLS-SEM Analysis Among Registered Nurses in Saudi Arabia* [Dataset]. Zenodo. https://doi.org/10.5281/zenodo.21345972. The repository includes the study questionnaire used for data collection. These materials are provided to facilitate transparency, reproducibility, and future research. The STROBE checklist for this cross-sectional study has been deposited in Zenodo and is available at: Othman AD, Hassan HC. STROBE Checklist [Dataset]. Zenodo. 2026. https://doi.org/10.5281/zenodo.20817498. Data are available under the terms of the Creative Commons Attribution 4.0 International (CC BY 4.0) licence.

## References

[ref1] Alfuqaha OA Alsharah HS Alqudah H : Work-life balance among nurses in healthcare settings: A cross-sectional study. *Nurs. Forum.* 2021;56(4):951–959.

[ref2] Ariasari NP Tjahjono HK : Leadership support, work-life balance, and employee well-being in healthcare organizations. *J. Health Organ. Manag.* 2024.

[ref3] Bakker AB Demerouti E : The job demands-resources model: State of the art. *J. Manag. Psychol.* 2007;22(3):309–328. 10.1108/02683940710733115

[ref22] Clark SC : Work/Family Border Theory: A New Theory of Work/Family Balance. *Hum. Relat.* 2000;53(6):747–770. 10.1177/0018726700536001

[ref4] Dall'Ora C Ball J Reinius M : Burnout in nursing: A theoretical review. *Hum. Resour. Health.* 2020;18:41. 10.1186/s12960-020-00469-9 PMC7273381 32503559

[ref5] Dousin O Collins N Kler BK : The relationship between work-life balance and employee well-being among healthcare professionals. *Int. J. Hum. Resour. Stud.* 2019;9(4):78–95.

[ref21] Elm E von Altman DG Egger M : The Strengthening the Reporting of Observational Studies in Epidemiology (STROBE) Statement: Guidelines for Reporting Observational Studies. *Lancet.* 2007;370(9596):1453–1457. 10.1016/S0140-6736(07)61602-X 18064739

[ref6] Fisher-McAuley G Stanton J Jolton J : Modelling the relationship between work-life balance and organizational outcomes. *Proceedings of the Annual Conference of the Society for Industrial and Organizational Psychology.* 2003.

[ref7] Fukuzaki K Imai K Watanabe M : Work-life balance and occupational well-being among nurses: Evidence from healthcare settings. *BMC Nurs.* 2021;20(1).

[ref8] Haar JM Russo M Suñe A : Outcomes of work-life balance on job satisfaction, life satisfaction and mental health: A study across seven cultures. *J. Vocat. Behav.* 2014;85(3):361–373. 10.1016/j.jvb.2014.08.010

[ref9] Hair JF Hult GTM Ringle CM : *A Primer on Partial Least Squares Structural Equation Modeling (PLS-SEM).* 3rd ed. Sage;2022.

[ref10] Haider S Jabeen S Ahmad J : Moderated mediation between work-life balance and employee performance: The role of psychological well-being. *J. Hum. Behav. Soc. Environ.* 2018;28(8).

[ref11] Helaly MA : Work-life balance, job stress, and performance among nurses in healthcare institutions. *BMC Nurs.* 2022;21(1).

[ref12] Hobfoll SE : Conservation of resources: A new attempt at conceptualizing stress. *Am. Psychol.* 1989;44(3):513–524. 10.1037/0003-066X.44.3.513 2648906

[ref13] Krejcie RV Morgan DW : Determining sample size for research activities. *Educ. Psychol. Meas.* 1970;30(3):607–610. 10.1177/001316447003000308

[ref14] Laschinger HKS Wong CA Grau AL : Authentic leadership, empowerment and burnout: Implications for nurse performance. *J. Nurs. Manag.* 2014;22(5):541–552. 10.1111/j.1365-2834.2012.01375.x 23405976

[ref15] Maslach C Leiter MP : *Burnout.* Wiley;2016.

[ref17] Robertson I Cooper C : Full engagement: The integration of employee engagement and psychological well-being. *Leadersh. Org. Dev. J.* 2010;31(4):324–336. 10.1108/01437731011043348

[ref18] Stankevičienė J Savanevičienė A Karaliūtė A : The impact of work-life balance on organizational sustainability and employee well-being. *Sustainability.* 2021;13(4).

[ref19] World Health Organization: *State of the world's nursing 2020: Investing in education, jobs and leadership.* World Health Organization;2020.

[ref20] Zhang Y Li X Wang H : Work-life balance, employee well-being, and nursing performance in tertiary healthcare settings. *BMC Nurs.* 2024;23(1).

