# Organizational and Psychological Determinants of Nursing Performance: A PLS-SEM Analysis Among Registered Nurses in Saudi Arabia

**DOI:** 10.12688/f1000research.186749.2

**Published:** 2026-10-10

**Authors:** Adawi Dalal Othman, Hafizah Che Hassan

**Affiliations:** 1 Lincoln University College, Petaling Jaya, Selangor, Malaysia

**Keywords:** work-life balance, nursing performance, employee well-being, stress reduction, nurses, PLS-SEM, occupational health, Saudi Arabia.

## Abstract

**Background:**

Maintaining high nursing performance is increasingly challenging in modern healthcare systems due to heavy workloads, workforce shortages, and growing psychological demands. Although work-life balance has been recognized as an important organizational resource, limited evidence has examined the statistical pathways linking work-life balance with nursing performance through employee well-being and stress reduction, particularly in Saudi Arabian healthcare settings. This study examined the associations among work-life balance, employee well-being, stress reduction, and nursing performance among registered nurses.

**Methods:**

A cross-sectional study was conducted among 427 registered nurses employed at The Medical City of Medina, Saudi Arabia. Proportionate stratification was used to ensure representation across clinical departments, and all eligible nurses within each stratum were invited to participate. Data were collected using structured self-administered questionnaires and analysed using Partial Least Squares Structural Equation Modelling (PLS-SEM). Direct and indirect associations were evaluated using a bootstrapping procedure with 5,000 resamples.

**Results:**

Work-life balance was positively associated with employee well-being (β = 0.885, *p* < 0.001), stress reduction (β = 0.888, *p* < 0.001), and nursing performance (β = 0.219, *p* < 0.001). Employee well-being (β = 0.320, *p* < 0.001) and stress reduction (β = 0.417, *p* < 0.001) were also positively associated with nursing performance. Statistically significant indirect associations were observed between work-life balance and nursing performance through employee well-being (β = 0.283, *p* < 0.001) and stress reduction (β = 0.371, *p* < 0.001), consistent with partial statistical mediation.

**Conclusions:**

Work-life balance was positively associated with nursing performance both directly and indirectly through employee well-being and stress reduction. The findings support the relevance of organizational conditions that address work-life balance, employee well-being, and occupational stress in nursing workforce management. Given the cross-sectional design, the observed associations and statistical mediation should not be interpreted as evidence of causal relationships. Longitudinal and multi-centre research is warranted to further examine these relationships across diverse healthcare settings.

## Introduction

Nurses constitute the largest professional workforce within healthcare systems and play a fundamental role in ensuring patient safety, healthcare quality, and continuity of care. The quality of nursing performance has become increasingly important as healthcare organizations worldwide continue to experience growing service demands, workforce shortages, increasing administrative responsibilities, and the long-term consequences of the COVID-19 pandemic. These challenges have intensified nurses’ workloads, increased occupational stress, and highlighted the importance of developing sustainable workforce strategies that promote both employee well-being and high-quality patient care ( World Health Organization, 2020; Dall’Ora et al., 2020; Zhang et al., 2024).

Among the organizational factors associated workforce sustainability, work-life balance (WLB) has received increasing attention in nursing research. Work-life balance refers to an individual’s ability to effectively balance professional responsibilities with personal and family life. In nursing practice, maintaining an appropriate work-life balance is particularly challenging because nurses frequently work rotating shifts, extended working hours, and emotionally demanding clinical environments. Previous studies have consistently demonstrated that inadequate work-life balance is associated with burnout, emotional exhaustion, reduced job satisfaction, increased turnover intention, and poorer quality of patient care. Conversely, supportive organizational policies, flexible work arrangements, and healthy work environments have been associated with greater employee engagement, improved workforce retention, and enhanced organizational effectiveness ( Fukuzaki et al., 2021; Helaly, 2022; Zhang et al., 2024; Ariasari & Tjahjono, 2024).

Although evidence consistently supports a positive association between work-life balance and employee outcomes, the pathways linking work-life balance with nursing performance remain incompletely understood. The Job Demands–Resources (JD-R) theory proposes that organizational resources enable employees to cope more effectively with demanding work environments, thereby improving motivation, engagement, and job performance ( Bakker & Demerouti, 2007). Similarly, the Conservation of Resources (COR) theory suggests that individuals seek to acquire, maintain, and protect valuable personal and occupational resources, whereas resource loss contributes to stress, diminished well-being, and impaired work performance ( Hobfoll, 1989). Within these theoretical perspectives, employee well-being and stress reduction may represent important organizational and psychological factors through which work-life balance is associated with nursing performance.

Despite the growing body of literature on work-life balance among healthcare professionals, several important knowledge gaps remain. Existing studies have primarily focused on direct associations between work-life balance and outcomes such as burnout, job satisfaction, and psychological well-being, whereas comparatively fewer studies have explored the combined roles of employee well-being and stress reduction in relation to nursing performance. Furthermore, most available evidence has been generated in Western countries and East Asia, with relatively limited research conducted in Middle Eastern healthcare systems despite differences in organizational structures, workforce composition, and sociocultural contexts ( Alfuqaha et al., 2021). Evidence from Saudi Arabian tertiary hospitals is particularly scarce, limiting the applicability of current knowledge to healthcare organizations operating within this setting.

Addressing these knowledge gaps is especially important in Saudi Arabia, where continuing healthcare reforms, increasing service demands, and the need to sustain a resilient nursing workforce require organizational strategies that effectively support nurses’ well-being and professional performance. A clearer understanding of the relationships among work-life balance, employee well-being, stress reduction, and nursing performance may provide healthcare administrators with context-specific evidence to inform organizational strategies for supporting workforce sustainability and maintaining high standards of patient care.

Therefore, this study aimed to examine the relationships among work-life balance, employee well-being, stress reduction, and nursing performance among registered nurses at The Medical City of Medina, Saudi Arabia. Guided by the Job Demands–Resources (JD-R) theory and the Conservation of Resources (COR) theory, the study sought to provide context-specific evidence regarding organizational and psychological factors associated with nursing performance in Saudi Arabian healthcare settings.

Specifically, the objectives of this study were: (1) to examine the association between work-life balance and nursing performance among registered nurses; (2) to examine the associations of work-life balance with employee well-being and stress reduction; (3) to examine the associations of employee well-being and stress reduction with nursing performance; and (4) to investigate the statistical mediating roles of employee well-being and stress reduction in the association between work-life balance and nursing performance.

The findings are expected to provide empirical evidence that may inform organizational strategies to support nurses’ well-being, occupational stress management, workforce sustainability, and the quality of healthcare delivery.

## Methods

### Study design and setting

This analytical cross-sectional study was conducted among registered nurses at The Medical City of Medina, Saudi Arabia, to examine the association between work-life balance and nursing performance and to investigate the potential statistical mediating roles of employee well-being and stress reduction. The study was reported in accordance with the Strengthening the Reporting of Observational Studies in Epidemiology (STROBE) Statement for cross-sectional studies ( von Elm et al., 2007).

Data were collected from nurses working in major clinical departments, including medical, surgical, emergency, intensive care, and outpatient services. Because the study aimed to examine associations among organizational and psychological factors at a single point in time, a cross-sectional design was considered appropriate.

### Participants and sampling

The target population comprised 520 registered nurses employed at The Medical City of Medina. Eligible participants were registered nurses who were employed full-time, had at least six months of clinical experience at the hospital, and provided written informed consent before participation. Nurses on extended leave during the study period and personnel without direct patient-care responsibilities were excluded.

The minimum required sample size was determined using the Krejcie and Morgan (1970) sample size table for finite populations at a 95% confidence level and a 5% margin of error, indicating that at least 222 participants were required. To ensure representation across clinical departments, proportionate stratification was used for participant recruitment, and all eligible nurses within each stratum were invited to participate. A total of 427 completed questionnaires were included in the final analysis, corresponding to an overall response rate of 82.1% (427/520).

### Study instrument

Data were collected using a structured self-administered questionnaire comprising five sections. The first section collected participants’ demographic characteristics, including age, sex, marital status, educational level, years of nursing experience, and work schedule.

The remaining sections assessed four latent constructs: work-life balance (8 items), employee well-being (8 items), stress reduction (8 items), and nursing performance (8 items). The work-life balance scale was adapted from Fisher-McAuley et al. (2003) and Haar et al. (2014). Employee well-being items were adapted from Clark (2000) and Robertson and Cooper (2010). Stress reduction was operationalized as nurses’ perceived ability to manage and cope with work-related stress, together with their perception that workplace conditions and organizational support help reduce psychological strain and facilitate recovery from work-related stress. The eight stress reduction items were developed based on concepts described by Maslach and Leiter (2016) and Helaly (2022). Nursing performance items were adapted from Laschinger et al. (2014) and Zhang et al. (2024). All questionnaire items were rated using a five-point Likert scale ranging from 1 (“strongly disagree”) to 5 (“strongly agree”), with higher scores indicating higher levels of the respective construct.

### Pilot testing

Prior to the main survey, the questionnaire was pilot tested among 20 registered nurses who were not included in the final study sample. The pilot study assessed questionnaire clarity, comprehensibility, and preliminary reliability. Internal consistency was evaluated using Cronbach’s alpha coefficients. All constructs demonstrated acceptable preliminary internal consistency, with alpha values exceeding the recommended threshold of 0.70. Cronbach’s alpha coefficients were 0.87 for work-life balance, 0.90 for employee well-being, 0.88 for stress reduction, and 0.86 for nursing performance, indicating satisfactory internal consistency of the measurement instrument.

### Data collection

Data were collected using both paper-based and electronic questionnaires. Departmental nurse managers assisted in distributing study invitations during departmental meetings and shift handovers. Before participation, eligible nurses received written information describing the study objectives, voluntary nature of participation, confidentiality safeguards, and their right to withdraw at any time without penalty. Questionnaires were completed anonymously. Anonymity and voluntary participation were emphasized to encourage honest responses and reduce potential social desirability during self-reporting. Responses were screened for completeness before analysis, and questionnaires containing substantial missing data were excluded.

### Statistical analysis

Data were analysed using SmartPLS version 4 (SmartPLS GmbH, Oststeinbek, Germany). Partial Least Squares Structural Equation Modelling (PLS-SEM) was used to examine the specified associations among work-life balance, employee well-being, stress reduction, and nursing performance, including the statistical mediating roles of employee well-being and stress reduction.

Descriptive statistics were calculated to summarize participants’ demographic characteristics and the distributions of the study constructs. Pearson correlation analysis was performed to examine bivariate associations among the four study constructs before evaluation of the PLS-SEM structural model.

The measurement model was evaluated by examining indicator reliability through outer loadings, internal consistency reliability using Cronbach’s alpha and composite reliability, and convergent validity using the average variance extracted (AVE).

The structural model was evaluated using standardized path coefficients (β), coefficients of determination (R ^2^), and the standardized root mean square residual (SRMR). The significance of direct and indirect associations was assessed using non-parametric bootstrapping with 5,000 resamples. Statistical significance was defined as p < 0.05.

## Results

### Participant characteristics

A total of 427 registered nurses completed the questionnaire and were included in the final analysis, corresponding to an overall response rate of 82.1% (427/520). Most participants were female, aged between 25 and 44 years, held a bachelor’s degree, and had between five and ten years of professional nursing experience ( Table 1).

**Table 1.** Demographic profile of respondents.

Demographic Variable	Category	Frequency (n)	Percentage (%)
**Gender**	Female	320	74.9%
	Male	107	25.1%
**Age**	Under 25	43	10.1%
	25–34	171	40.0%
	35–44	128	30.0%
	45–54	64	15.0%
	55+	21	4.9%
**Marital Status**	Single	128	30.0%
	Married	256	60.0%
	Divorced	34	8.0%
	Widowed	9	2.0%
**Education**	Diploma	85	19.9%
	Bachelor	278	65.1%
	Master	55	12.9%
	Doctorate	9	2.1%
**Experience**	< 5 years	85	19.9%
	5–10 years	171	40.0%
	11–15 years	107	25.1%
	> 15 years	64	15.0%
**Work Schedule**	Day	171	40.0%
	Night	85	19.9%
	Rotational	171	40.0%

### Pearson correlation analysis

Pearson correlation analysis Pearson correlation analysis showed statistically significant positive bivariate associations among all four study constructs ( Table 2). Work-life balance was positively correlated with employee well-being (r = 0.652, p < 0.01), stress reduction (r = 0.584, p < 0.01), and nursing performance (r = 0.621, p < 0.01). Employee well-being was positively correlated with stress reduction (r = 0.610, p < 0.01) and nursing performance (r = 0.685, p < 0.01), while stress reduction was also positively correlated with nursing performance (r = 0.553, p < 0.01).

**Table 2.** Pearson correlations among study constructs.

Construct	WLB	EWB	SR	NP
Work-Life Balance (WLB)	1.000	0.652	0.584	0.621
Employee Well-Being (EWB)	0.652	1.000	0.610	0.685
Stress Reduction (SR)	0.584	0.610	1.000	0.553
Nursing Performance (NP)	0.621	0.685	0.553	1.000

**Note:** p < 0.01, two-tailed. WLB = work-life balance; EWB = employee well-being; SR = stress reduction; NP = nursing performance.

### Measurement model assessment

Measurement model assessment The measurement model demonstrated satisfactory indicator reliability, internal consistency reliability, and convergent validity ( Table 3). All 32 indicators had outer loadings of ≥0.70, ranging from 0.707 to 0.808. For work-life balance, outer loadings ranged from 0.712 to 0.771; for employee well-being, from 0.707 to 0.760; for stress reduction, from 0.734 to 0.808; and for nursing performance, from 0.709 to 0.786. Cronbach’s alpha values ranged from 0.876 to 0.893, while composite reliability values ranged from 0.902 to 0.914. Average Variance Extracted (AVE) values ranged from 0.535 to 0.572, exceeding the recommended threshold of 0.50 and supporting convergent validity.

**Table 3.** Measurement model assessment.

Construct	Indicator	Outer loading	Cronbach’s α	Composite reliability	AVE
Work-Life Balance	WLB1	0.726	0.879	0.904	0.542
	WLB2	0.771			
	WLB3	0.722			
	WLB4	0.725			
	WLB5	0.743			
	WLB6	0.712			
	WLB7	0.743			
	WLB8	0.748			
Employee Well-Being	EWB1	0.709	0.876	0.902	0.535
	EWB2	0.730			
	EWB3	0.707			
	EWB4	0.760			
	EWB5	0.740			
	EWB6	0.716			
	EWB7	0.742			
	EWB8	0.746			
Stress Reduction	SR1	0.755	0.893	0.914	0.572
	SR2	0.772			
	SR3	0.736			
	SR4	0.760			
	SR5	0.734			
	SR6	0.740			
	SR7	0.740			
	SR8	0.808			
Nursing Performance	NP1	0.726	0.882	0.906	0.548
	NP2	0.755			
	NP3	0.747			
	NP4	0.737			
	NP5	0.740			
	NP6	0.786			
	NP7	0.709			
	NP8	0.718			

### Structural model assessment

The structural model showed high explained variance for the endogenous constructs ( Table 4). Work-life balance accounted for 78.3% of the variance in employee well-being (R ^2^ = 0.783) and 78.9% of the variance in stress reduction (R ^2^ = 0.789). Work-life balance, employee well-being, and stress reduction jointly accounted for 84.7% of the variance in nursing performance (R ^2^ = 0.847). The standardized root mean square residual was 0.044.

**Table 4.** Coefficients of determination and model fit

Endogenous Construct	R ^**2**^
Employee Well-Being	0.783
Stress Reduction	0.789
Nursing Performance	0.847

### Direct associations

The bootstrapping analysis indicated statistically significant positive associations for all prespecified structural paths ( Table 5). Work-life balance was positively associated with employee well-being (β = 0.885, t = 87.659, p < 0.001), stress reduction (β = 0.888, t = 94.286, p < 0.001), and nursing performance (β = 0.219, t = 4.653, p < 0.001). Employee well-being was also positively associated with nursing performance (β = 0.320, t = 6.878, p < 0.001), as was stress reduction (β = 0.417, t = 8.990, p < 0.001).

**Table 5.** Direct associations between study constructs.

Hypothesis	Path	β	t-value	p-value	Decision
H1	WLB → EWB	0.885	87.659	<0.001	Supported
H2	WLB → SR	0.888	94.286	<0.001	Supported
H3	WLB → NP	0.219	4.653	<0.001	Supported
H4	EWB → NP	0.320	6.878	<0.001	Supported
H5	SR → NP	0.417	8.990	<0.001	Supported

Note: WLB = work-life balance; EWB = employee well-being; SR = stress reduction; NP = nursing performance; β = standardized path coefficient.

### Mediation analysis

The mediation analysis indicated statistically significant indirect associations between work-life balance and nursing performance through employee well-being and stress reduction ( Table 6). The indirect association through employee well-being was statistically significant (β = 0.283, t = 6.904, p < 0.001), as was the indirect association through stress reduction (β = 0.371, t = 8.918, p < 0.001). Because the corresponding direct association between work-life balance and nursing performance remained statistically significant, the results were consistent with partial statistical mediation.

**Table 6.** Indirect associations and statistical mediation.

Indirect Path	β	t-value	p-value	Interpretation
Work-Life Balance → Employee Well-Being → Nursing Performance	0.283	6.904	<0.001	Significant partial statistical mediation
Work-Life Balance → Stress Reduction → Nursing Performance	0.371	8.918	<0.001	Significant partial statistical mediation

## Discussion

The present study examined the associations among work-life balance, employee well-being, stress reduction, and nursing performance among registered nurses working in a tertiary healthcare institution in Saudi Arabia using Partial Least Squares Structural Equation Modelling (PLS-SEM). The findings showed statistically significant positive associations between work-life balance and employee well-being, stress reduction, and nursing performance. Employee well-being and stress reduction were also positively associated with nursing performance, while statistically significant indirect associations were observed through both employee well-being and stress reduction. Because the study used a cross-sectional design, these findings should be interpreted as statistical associations rather than evidence of causal effects.

The significant positive association between work-life balance and nursing performance is consistent with previous evidence indicating that nurses who experience greater balance between professional and personal responsibilities may report better work engagement, productivity, and quality of care. Nurses routinely encounter substantial workloads, rotating shifts, emotional labour, and administrative responsibilities, all of which may be associated with fatigue and difficulties in maintaining sustained clinical functioning when opportunities for recovery are limited. Conversely, organizational conditions that facilitate work-life balance may be associated with greater resilience, professional commitment, and sustained job performance ( Fukuzaki et al., 2021; Zhang et al., 2024). These findings suggest that work-life balance is relevant to nursing performance within the studied setting, although the cross-sectional design does not establish the direction or causality of these relationships.

Stress reduction showed a statistically significant positive association with nursing performance (β = 0.417). This association indicates that nurses reporting greater perceived ability to manage work-related stress, supportive workplace conditions, and psychological recovery also tended to report higher nursing performance. The finding is consistent with previous evidence linking occupational stress management, supportive leadership, and healthy work environments with workforce outcomes ( Stankevičienė et al., 2021; Zhang et al., 2024). However, the magnitude of this coefficient should not be interpreted as evidence that stress reduction has a greater causal effect on performance than the other variables examined.

The relatively high path coefficients observed for the associations between work-life balance and employee well-being (β = 0.885) and between work-life balance and stress reduction (β = 0.888), together with the high explained variance for the endogenous constructs, warrant cautious interpretation. These coefficients indicate strong statistical associations within the specified PLS-SEM model, but they should not be interpreted as evidence of causal effects. The relatively high explained variance may partly reflect conceptual proximity among the constructs, particularly because the measures capture related aspects of nurses’ work experiences and psychological responses. Because numerical HTMT and structural VIF diagnostics were not available in the archived analysis output, the possibility of construct overlap or collinearity cannot be evaluated as comprehensively as would be desirable. Future analyses should explicitly report these diagnostics to further assess discriminant validity and collinearity.

Employee well-being was also positively associated with nursing performance (β = 0.320) and showed a statistically significant indirect association between work-life balance and nursing performance. This finding is consistent with previous evidence suggesting that psychologically healthy nurses may be more engaged, resilient, and able to maintain professional functioning under demanding workplace conditions ( Dousin et al., 2019; Haider et al., 2018). In the present study, employee well-being therefore represents one statistical pathway through which work-life balance was associated with nursing performance. However, the observed indirect association should be interpreted as statistical mediation rather than evidence of a temporally established causal mechanism.

The mediation analysis provides additional insight into the statistical pathways linking work-life balance with nursing performance. Both employee well-being and stress reduction showed statistically significant indirect associations, while the direct association between work-life balance and nursing performance remained significant. This pattern was consistent with partial statistical mediation. The findings suggest that the association between work-life balance and nursing performance may involve psychological dimensions related to well-being and perceived stress management, while also indicating that other factors not included in the model may account for part of the observed variation in nursing performance.

The findings can be interpreted through the **Job Demands–Resources (JD-R) theory** ( Bakker & Demerouti, 2007). From a JD-R perspective, work-life balance can be considered a work-related condition that may function as a resource by facilitating recovery and helping employees manage the demands associated with clinical work. Within this framework, the observed associations between work-life balance, employee well-being, stress reduction, and nursing performance are consistent with the proposition that access to supportive work resources is related to more favourable psychological and occupational outcomes. The present findings therefore extend the application of the JD-R framework to a nursing context in which work demands include workload, shift work, emotional labour, and competing professional and personal responsibilities.

The **Conservation of Resources (COR) theory** ( Hobfoll, 1989) provides a complementary but distinct perspective. Whereas JD-R emphasizes the relationship between work demands, work resources, and occupational outcomes, COR focuses on individuals’ motivation to acquire, maintain, and protect valued resources. From a COR perspective, difficulties in balancing work and personal responsibilities may be associated with perceived resource depletion, whereas conditions that facilitate recovery and stress management may be associated with the preservation of psychological and emotional resources. The observed associations with employee well-being and stress reduction are therefore compatible with the COR proposition that resource preservation is relevant to psychological functioning. Taken together, JD-R helps explain the role of work-related resources within the occupational environment, whereas COR provides a complementary explanation centred on the preservation and depletion of personal resources.

An additional contribution of this study is the provision of evidence from a tertiary healthcare setting in Saudi Arabia. Previous research examining work-life balance and nursing outcomes has been conducted across diverse geographical and organizational contexts, while evidence from Saudi Arabian healthcare settings remains comparatively limited. Differences in healthcare systems, workforce composition, organizational culture, and sociocultural expectations may shape nurses’ experiences of work-life balance and occupational stress. The present findings therefore provide context-specific evidence regarding the associations among work-life balance, psychological factors, and nursing performance in a Saudi Arabian tertiary healthcare setting. They also provide an empirical context in which the JD-R and COR perspectives can be considered in relation to nursing workforce outcomes.

From a practical perspective, the findings suggest that healthcare organizations may benefit from workforce strategies that address work-life balance, employee well-being, and occupational stress simultaneously. Potential organizational approaches include flexible or predictable work scheduling, adequate staffing, supportive leadership, access to psychological support, and structured workplace wellness initiatives. These approaches are consistent with the observed associations in the present study, although their effectiveness cannot be established from the current cross-sectional design. Organizational decision-making should therefore consider these findings alongside local workforce needs, existing institutional policies, and evidence from longitudinal or intervention research.

### Strengths and limitations

This study has several strengths. First, it included a relatively large sample of 427 registered nurses recruited from multiple clinical departments within a tertiary healthcare institution. Second, the study was informed by two complementary theoretical frameworks, the Job Demands–Resources (JD-R) theory and Conservation of Resources (COR) theory, providing distinct perspectives on work-related resources and the preservation of psychological resources. Third, the use of PLS-SEM enabled the simultaneous examination of direct and indirect statistical associations among work-life balance, employee well-being, stress reduction, and nursing performance. Finally, the study employed a structured multi-item questionnaire and reported indicator loadings, internal consistency reliability, composite reliability, convergent validity, explained variance, model fit, and bootstrapped direct and indirect associations.

Several limitations should also be acknowledged. First, the cross-sectional design prevents determination of temporal ordering and causal relationships among work-life balance, employee well-being, stress reduction, and nursing performance. The statistical mediation observed in this study should therefore be interpreted as an indirect statistical association rather than evidence of a causal mechanism. Longitudinal or prospective studies are needed to examine temporal relationships among these constructs.

Second, all major study constructs, including nursing performance, were measured using self-administered questionnaires. This introduces the possibility of common method variance and social desirability bias. Although anonymous participation and confidentiality procedures were used to encourage honest responses, these procedural measures cannot fully eliminate such bias. No independent supervisor-rated or objective measure of nursing performance was obtained. Future studies should therefore consider combining self-reported measures with supervisor-rated performance, objective performance indicators, or longitudinal measurements to reduce reliance on a single measurement source.

Third, the data were collected from a single tertiary healthcare institution in Saudi Arabia, which may limit the transferability of the findings to other hospitals, healthcare organizations, or countries. Differences in organizational structures, staffing models, workforce characteristics, and sociocultural contexts may influence the observed associations.

Fourth, although the model explained a substantial proportion of the variance in nursing performance, other potentially relevant factors were not included, including leadership style, organizational culture, psychological safety, staffing adequacy, resilience, burnout, and work engagement. These factors may also be associated with nursing performance and could provide additional explanatory value in future models.

Finally, the present study reports the PLS-SEM assessment based on the model evaluation metrics available from the analysis, including indicator loadings, internal consistency reliability, convergent validity, explained variance (R ^2^), model fit (SRMR), and bootstrapped direct and indirect associations. Complementary diagnostics such as HTMT, structural VIF, f ^2^, and Q ^2^ may provide additional information regarding discriminant validity, collinearity, effect sizes, and predictive relevance and should be considered in future analyses.

### Future research

Future research should employ longitudinal or prospective study designs to examine the temporal relationships among work-life balance, employee well-being, stress reduction, and nursing performance. Multi-centre studies involving different healthcare organizations and geographical settings would also help assess the transferability of the findings beyond a single tertiary healthcare institution in Saudi Arabia.

Future studies should incorporate multiple sources of performance assessment, including supervisor ratings and objective performance indicators where feasible, to reduce potential common method bias associated with self-reported outcomes. Researchers could also examine additional organizational and psychological factors, such as transformational leadership, organizational support, psychological safety, staffing adequacy, burnout, resilience, and work engagement, to determine whether these factors provide additional explanatory value beyond the constructs examined in the present study.

## Conclusion

This study found statistically significant positive associations among work-life balance, employee well-being, stress reduction, and nursing performance among registered nurses in a tertiary healthcare institution in Saudi Arabia. Work-life balance was positively associated with employee well-being, stress reduction, and nursing performance, while employee well-being and stress reduction were also positively associated with nursing performance. Statistically significant indirect associations were observed through both employee well-being and stress reduction, with the findings being consistent with partial statistical mediation. Given the cross-sectional design, these findings should be interpreted as associations rather than causal relationships.

The findings provide empirical support for considering both work-related resources and psychological resource preservation when examining nursing performance, consistent with complementary perspectives from the Job Demands–Resources and Conservation of Resources theories. The relatively high associations between work-life balance and the psychological constructs also highlight the importance of cautious interpretation and further evaluation of construct relationships in future research.

From a practical perspective, healthcare organizations may consider integrated workforce strategies addressing work-life balance, employee well-being, and occupational stress, including supportive scheduling practices, adequate staffing, supportive leadership, and accessible psychological support. These strategies may provide a useful organizational framework for supporting nurses’ well-being and workforce sustainability, although their effectiveness should be evaluated through longitudinal and intervention research.

Future multi-centre and longitudinal studies should examine the temporal relationships among these constructs, incorporate objective or supervisor-rated measures of nursing performance, and evaluate additional organizational and psychological factors that may contribute to nursing performance across diverse healthcare settings.

### Ethical considerations

This study was conducted in accordance with the ethical principles of the Declaration of Helsinki. Ethical approval was obtained from the Research Ethics Committee of Lincoln University College, prior to the commencement of the study (Approval No. LUC/MKT/SP/007/223). Written informed consent was obtained from all participants before data collection. Participants were informed about the purpose of the study, the voluntary nature of participation, their right to withdraw at any time without penalty, and the confidentiality of their responses. All data were de-identified prior to analysis and used solely for research purposes.

## Data Availability

The de-identified dataset supporting the findings of this study is available from Zenodo: Adawi Dalal, O., & Hafizah Che, H. (2026). *Data Sheet: Work-Life Balance and Nursing Performance Among Registered Nurses in Saudi Arabia* [Dataset]. Zenodo. https://doi.org/10.5281/zenodo.21293834. The dataset contains anonymized questionnaire responses and does not include personally identifiable information. The study materials supporting this research are available from Zenodo: Adawi Dalal, O., & Hafizah Che, H. (2026). *Extended Data for: Organizational and Psychological Determinants of Nursing Performance: A PLS-SEM Analysis Among Registered Nurses in Saudi Arabia* [Dataset]. Zenodo. https://doi.org/10.5281/zenodo.21345972. The repository includes the study questionnaire used for data collection. These materials are provided to facilitate transparency, reproducibility, and future research. The STROBE checklist for this cross-sectional study has been deposited in Zenodo and is available at: Othman AD, Hassan HC. STROBE Checklist [Dataset]. Zenodo. 2026. https://doi.org/10.5281/zenodo.20817498. Data are available under the terms of the Creative Commons Attribution 4.0 International (CC BY 4.0) licence.

## References

[ref1] Alfuqaha OA Alsharah HS Alqudah H : Work-life balance among nurses in healthcare settings: A cross-sectional study. *Nurs. Forum.* 2021;56(4):951–959.

[ref2] Ariasari NP Tjahjono HK : Leadership support, work-life balance, and employee well-being in healthcare organizations. *J. Health Organ. Manag.* 2024.

[ref3] Bakker AB Demerouti E : The job demands-resources model: State of the art. *J. Manag. Psychol.* 2007;22(3):309–328. 10.1108/02683940710733115

[ref22] Clark SC : Work/Family Border Theory: A New Theory of Work/Family Balance. *Hum. Relat.* 2000;53(6):747–770. 10.1177/0018726700536001

[ref4] Dall’Ora C Ball J Reinius M : Burnout in nursing: A theoretical review. *Hum. Resour. Health.* 2020;18:41. 10.1186/s12960-020-00469-9 PMC7273381 32503559

[ref5] Dousin O Collins N Kler BK : The relationship between work-life balance and employee well-being among healthcare professionals. *Int. J. Hum. Resour. Stud.* 2019;9(4):78–95.

[ref21] Elm E von Altman DG Egger M : The Strengthening the Reporting of Observational Studies in Epidemiology (STROBE) Statement: Guidelines for Reporting Observational Studies. *Lancet.* 2007;370(9596):1453–1457. 10.1016/S0140-6736(07)61602-X 18064739

[ref6] Fisher-McAuley G Stanton J Jolton J : Modelling the relationship between work-life balance and organizational outcomes. *Proceedings of the Annual Conference of the Society for Industrial and Organizational Psychology.* 2003.

[ref7] Fukuzaki K Imai K Watanabe M : Work-life balance and occupational well-being among nurses: Evidence from healthcare settings. *BMC Nurs.* 2021;20(1).

[ref8] Haar JM Russo M Suñe A : Outcomes of work-life balance on job satisfaction, life satisfaction and mental health: A study across seven cultures. *J. Vocat. Behav.* 2014;85(3):361–373. 10.1016/j.jvb.2014.08.010

[ref9] Hair JF Hult GTM Ringle CM : *A Primer on Partial Least Squares Structural Equation Modeling (PLS-SEM).* 3rd ed. Sage;2022.

[ref10] Haider S Jabeen S Ahmad J : Moderated mediation between work-life balance and employee performance: The role of psychological well-being. *J. Hum. Behav. Soc. Environ.* 2018;28(8).

[ref11] Helaly MA : Work-life balance, job stress, and performance among nurses in healthcare institutions. *BMC Nurs.* 2022;21(1).

[ref12] Hobfoll SE : Conservation of resources: A new attempt at conceptualizing stress. *Am. Psychol.* 1989;44(3):513–524. 10.1037/0003-066X.44.3.513 2648906

[ref13] Krejcie RV Morgan DW : Determining sample size for research activities. *Educ. Psychol. Meas.* 1970;30(3):607–610. 10.1177/001316447003000308

[ref14] Laschinger HKS Wong CA Grau AL : Authentic leadership, empowerment and burnout: Implications for nurse performance. *J. Nurs. Manag.* 2014;22(5):541–552. 10.1111/j.1365-2834.2012.01375.x 23405976

[ref15] Maslach C Leiter MP : *Burnout.* Wiley;2016.

[ref17] Robertson I Cooper C : Full engagement: The integration of employee engagement and psychological well-being. *Leadersh. Org. Dev. J.* 2010;31(4):324–336. 10.1108/01437731011043348

[ref18] Stankevičienė J Savanevičienė A Karaliūtė A : The impact of work-life balance on organizational sustainability and employee well-being. *Sustainability.* 2021;13(4).

[ref19] World Health Organization: *State of the world’s nursing 2020: Investing in education, jobs and leadership.* World Health Organization;2020.

[ref20] Zhang Y Li X Wang H : Work-life balance, employee well-being, and nursing performance in tertiary healthcare settings. *BMC Nurs.* 2024;23(1).

