# Supplementary material for: Organizational and Psychological Determinants of Nursing Performance: A PLS-SEM Analysis Among Registered Nurses in Saudi Arabia
Source: F1000Res. 2026 Oct 10;15:1530. Originally published 2026 Sep 11. [Version 2] doi: 10.12688/f1000research.186749.2 (PMC13684030; doi:10.12688/f1000research.186749.2)
Supplement: Peer review history

# Reviewer report: Reviewer response for version 2

**DOI:** 10.5256/f1000research.211914.r528631

**Published:** 2026-10-20

**Authors:** Farah Chalida Hanoum

**Affiliations:** 1 Universitas PGRI Semarang, Semarang, Central Java, Indonesia

the revision have completed, **approved for indexing**

Is the work clearly and accurately presented and does it cite the current literature?

Partly

If applicable, is the statistical analysis and its interpretation appropriate?

Partly

Are all the source data underlying the results available to ensure full reproducibility?

Yes

Is the study design appropriate and is the work technically sound?

Partly

Are the conclusions drawn adequately supported by the results?

Partly

Are sufficient details of methods and analysis provided to allow replication by others?

Partly

Reviewer Expertise:

Human Resource Management, Organizational Behavior, Nursing Management, Employee Well-Being, Work-Life Balance, Occupational Stress, and PLS-SEM.

I confirm that I have read this submission and believe that I have an appropriate level of expertise to confirm that it is of an acceptable scientific standard.

# Reviewer report: Reviewer response for version 2

**DOI:** 10.5256/f1000research.211914.r529831

**Published:** 2026-10-15

**Authors:** Suryanti Chan

**Affiliations:** 1 Universitas Dian Nuswantoro, Semarang, Indonesia

The manuscript entitled *“Organizational and Psychological Determinants of Nursing Performance: A PLS-SEM Analysis Among Registered Nurses in Saudi Arabia”* addresses an important and relevant topic in nursing management and healthcare workforce research. The study has the potential to contribute to a better understanding of the organizational and psychological factors associated with nursing performance. Overall, the manuscript is clearly presented, addresses a relevant research question, and appears to be supported by appropriate literature. The study design and general methodological approach are suitable for the research objectives.

 I recommend that the authors consider the following points:

1. Clarification of the theoretical contribution. The authors are encouraged to clarify how the findings contribute to existing theories and literature on organizational and psychological determinants of nursing performance. The Discussion could more explicitly explain the implications of the findings for nursing management and healthcare organizations in Saudi Arabia.
2. Statistical reporting and interpretation **.** The authors should ensure that the PLS-SEM procedures and their results are reported clearly and consistently. In particular, the reporting of measurement-model reliability and validity, path coefficients, bootstrapping results, and relevant model evaluation measures should be checked for completeness, where applicable. I would also welcome a brief clarification of the rationale for using PLS-SEM in relation to the study objectives. These suggestions are intended to improve transparency and should not be interpreted as a conclusion that the current statistical analysis is incorrect. Where necessary, a statistical expert may assist in verifying the interpretation of the results.
3. Interpretation of associations. The authors should ensure that the wording of the Results, Discussion, and Conclusions accurately reflects the study design. If the data are cross-sectional, relationships should generally be interpreted as associations rather than definitive causal effects. The conclusions should remain proportionate to the evidence presented.
4. Limitations and generalizability. The limitations section could briefly address the potential influence of self-reported measures, if applicable, and the extent to which the findings may be generalized beyond the participating nurses and healthcare settings in Saudi Arabia.
5. Practical implications **.** The manuscript would benefit from a concise explanation of how the findings might inform nursing leadership, organizational support, staff development, or interventions aimed at improving nursing performance. These implications should be directly linked to the significant findings of the study.
6. Final consistency check. The authors should verify the consistency of statistical values, terminology, tables, figures, and interpretations throughout the manuscript. The data availability statement should also accurately describe how the underlying data can be accessed, subject to applicable ethical and confidentiality requirements.

Overall, I find the manuscript relevant and potentially valuable. The points raised above are intended to refine the presentation and interpretation of the study rather than require substantial methodological changes. I believe that addressing these relatively focused issues would improve the manuscript's clarity, transparency, and practical relevance.

Is the work clearly and accurately presented and does it cite the current literature?

Yes

If applicable, is the statistical analysis and its interpretation appropriate?

I cannot comment. A qualified statistician is required.

Are all the source data underlying the results available to ensure full reproducibility?

Yes

Is the study design appropriate and is the work technically sound?

Yes

Are the conclusions drawn adequately supported by the results?

Yes

Are sufficient details of methods and analysis provided to allow replication by others?

Yes

Reviewer Expertise:

public health, healthcare quality assurance, health promotion, medical education

I confirm that I have read this submission and believe that I have an appropriate level of expertise to confirm that it is of an acceptable scientific standard.

## References

[1] A Primer on Partial Least Squares Structural Equation Modeling (PLS-SEM). *https://www.researchgate.net/publication/354331182_A_Primer_on_Partial_Least_Squares_Structural_Equation_Modeling_PLS-SEM* .

# Reviewer report: Reviewer response for version 1

**DOI:** 10.5256/f1000research.206113.r521588

**Published:** 2026-10-01

**Authors:** Farah Chalida Hanoum

**Affiliations:** 1 Universitas PGRI Semarang, Semarang, Central Java, Indonesia

**Summary and overall assessment**

 This manuscript examines the relationships among work-life balance, employee well-being, stress reduction, and nursing performance among 427 registered nurses working at The Medical City of Medina, Saudi Arabia. The study uses a cross-sectional design and proportionate stratified sampling, with data analysed using Partial Least Squares Structural Equation Modelling (PLS-SEM) and 5,000 bootstrap resamples. The results indicate that work-life balance is positively associated with employee well-being, stress reduction, and nursing performance. Employee well-being and stress reduction are also positively associated with nursing performance, while both variables significantly mediate the relationship between work-life balance and nursing performance. Stress reduction has the strongest direct association with nursing performance.

 The topic is relevant to healthcare management and nursing workforce sustainability, particularly given the challenges associated with workload, occupational stress, and employee well-being. The manuscript also provides useful context-specific evidence from Saudi Arabia, where the authors identify a relative lack of empirical research on these relationships. Overall, the manuscript has a clear research objective, an appropriate quantitative approach, and potentially useful theoretical and practical implications.

**Major Comments**

**1. Clarification of the sampling procedure**

 The manuscript states that proportionate stratified random sampling was used, but it subsequently indicates that “all eligible nurses within each stratum were invited to participate.” These descriptions may imply different sampling procedures. The authors should clarify whether nurses were randomly selected within each clinical department or whether all eligible nurses in each stratum were invited. Providing the number of eligible nurses and the number sampled/invited from each stratum would improve transparency and reproducibility.

**2. Clarification of the very high structural path coefficients**

 The relationships between work-life balance and employee well-being (β = 0.885) and between work-life balance and stress reduction (β = 0.888) are particularly strong. These coefficients, together with the high R² values for employee well-being (0.783), stress reduction (0.789), and nursing performance (0.847), deserve additional discussion.

 The authors should report and discuss the HTMT and VIF results in greater detail, particularly because conceptual overlap between work-life balance, well-being, and stress-related constructs could potentially contribute to the very strong relationships. Although the manuscript states that discriminant validity and multicollinearity were assessed, the corresponding numerical results are not presented in the tables. Reporting these values would allow readers to better evaluate the distinctiveness of the constructs.

**3. Further justification and clarification of the “stress reduction” construct**

 The operationalization of stress reduction requires further explanation. The manuscript indicates that the nine stress-reduction items were developed based on Maslach and Leiter (2016) and Helaly (2022). However, it would be useful to clarify precisely what “stress reduction” represents as a latent construct. For example, does it measure lower perceived occupational stress, employees’ ability to manage stress, or perceptions of organizational mechanisms that reduce stress? Clearer conceptual and operational definitions would strengthen the theoretical consistency of the model.

**4. More detailed reporting of measurement model results**

 The manuscript reports Cronbach’s alpha, composite reliability, and AVE, and states that all outer loadings exceeded 0.70. However, the individual indicator loadings are not presented. Since the study uses several multi-item constructs, providing a supplementary table containing the individual item loadings, indicator reliability, HTMT values, and VIF values would substantially improve transparency and reproducibility.

 The availability of the questionnaire through the Extended Data repository is a strength, but linking the specific items to each construct in the main manuscript or supplementary material would make the measurement model easier to evaluate.

**5. Caution regarding causal language**

 Because the study uses a cross-sectional design, causal relationships cannot be established. The manuscript appropriately acknowledges this limitation, but terms such as “determinants,” “influenced,” and “contributes to” should be used cautiously throughout the manuscript. It would be more appropriate to consistently describe the findings as associations or relationships unless causal evidence is available. The limitation concerning causal inference is already acknowledged by the authors and should also be reflected consistently in the Abstract, Discussion, and Conclusion.

**6. Common method and self-report bias**

 All major constructs appear to have been measured using self-administered questionnaires. Although the authors acknowledge the possibility of common method and social desirability bias, this issue deserves further consideration because both predictors and the outcome, including nursing performance, were measured from the same source.

 The authors could strengthen the discussion by explaining whether any procedural or statistical remedies for common method bias were applied. Future research could also consider supervisor-rated nursing performance, objective performance indicators, or longitudinal data to reduce dependence on self-reported outcomes.

1. The manuscript would benefit from more explicit reporting of the VIF, f², and Q² results because these analyses are stated to have been conducted but their numerical results are not presented in the Results section.
2. Pearson correlation analysis is described in the Methods section, but the corresponding correlation results are not reported. The authors should either provide the correlation matrix or clarify why these results are not presented.
3. The authors should clarify whether the reported response rate of 82.1% was calculated from all 520 nurses in the target population or from the number of eligible nurses who were actually invited to participate.
4. The manuscript provides useful information regarding ethical approval, informed consent, confidentiality, and anonymization. These details are appropriate and strengthen the ethical transparency of the study.
5. The availability of the anonymized dataset, questionnaire, and STROBE checklist through Zenodo is a positive feature that supports transparency and reproducibility.
6. The Discussion is generally well connected to the JD-R and Conservation of Resources theories. However, the authors could further explain the distinct contribution of each theory rather than mainly using both theories to provide a general explanation of the findings.

**Strengths**

 The main strengths of the manuscript include the relatively large sample of 427 registered nurses, the inclusion of nurses from multiple clinical departments, the use of established theoretical frameworks, and the application of PLS-SEM to examine both direct and mediation relationships. The study also addresses an under-researched Saudi Arabian healthcare context and provides potentially useful implications for healthcare workforce management.

 Another important strength is the authors’ commitment to research transparency. The manuscript reports ethical approval and informed consent procedures and provides access to the anonymized dataset, questionnaire, and reporting checklist through Zenodo.

**Conclusion**

 Overall, this is a relevant and potentially valuable study that contributes empirical evidence concerning the relationships between work-life balance, employee well-being, stress reduction, and nursing performance in a Saudi Arabian tertiary healthcare setting. The manuscript has a clear conceptual structure and reports statistically significant direct and indirect relationships. However, clarification of the sampling procedure, stronger reporting of the measurement and structural model diagnostics, further justification of the stress-reduction construct, and more cautious interpretation of causal implications would improve the methodological transparency and scientific contribution of the article. Addressing these points would strengthen the manuscript and make its findings more informative for researchers and healthcare practitioners.

Is the work clearly and accurately presented and does it cite the current literature?

Partly

If applicable, is the statistical analysis and its interpretation appropriate?

Partly

Are all the source data underlying the results available to ensure full reproducibility?

Yes

Is the study design appropriate and is the work technically sound?

Partly

Are the conclusions drawn adequately supported by the results?

Partly

Are sufficient details of methods and analysis provided to allow replication by others?

Partly

Reviewer Expertise:

Human Resource Management, Organizational Behavior, Nursing Management, Employee Well-Being, Work-Life Balance, Occupational Stress, and PLS-SEM.

I confirm that I have read this submission and believe that I have an appropriate level of expertise to state that I do not consider it to be of an acceptable scientific standard, for reasons outlined above.

## References

[1] : Organizational and Psychological Determinants of Nursing Performance: A PLS-SEM Analysis Among Registered Nurses in Saudi Arabia. *F1000Research* .2026;15: 10.12688/f1000research.186749.1 10.12688/f1000research.186749.1

# Response

**Published:** 2026-10-02

**Authors:** Adawi Dalal Othman

**Affiliations:** Faculty of Nursing, Lincoln University College, Petaling Jaya, Selangor, Malaysia

Response to Reviewer

 We sincerely thank the reviewer for the careful and constructive evaluation of our manuscript. We greatly appreciate the detailed comments regarding the sampling procedure, measurement model, structural model assessment, interpretation of the findings, theoretical contribution, and reporting transparency. We have carefully considered each point and substantially revised the manuscript accordingly. In particular, we have clarified the sampling procedure, expanded the reporting of indicator-level measurement results, added the Pearson correlation results, refined the operational definition of stress reduction, consistently replaced causal language with association-based terminology, expanded the discussion of common method bias, and further distinguished the contributions of the Job Demands–Resources and Conservation of Resources theories. Our detailed responses are provided below.

 Major Comment 1. Clarification of the sampling procedure

 Reviewer comment: The manuscript states that proportionate stratified random sampling was used, but it subsequently indicates that “all eligible nurses within each stratum were invited to participate.” These descriptions may imply different sampling procedures. The authors should clarify whether nurses were randomly selected within each clinical department or whether all eligible nurses in each stratum were invited. Providing the number of eligible nurses and the number sampled/invited from each stratum would improve transparency and reproducibility.

 Response: Thank you for identifying this inconsistency. We agree that the original description could imply that individual nurses were randomly selected within each clinical department. In fact, the target population comprised 520 registered nurses, all of whom met the eligibility criteria and were invited to participate. Proportionate stratification was used to ensure representation across clinical departments, rather than to randomly select individual nurses within each stratum.

 We have therefore removed the term “random sampling” and clarified the recruitment procedure in the revised manuscript. The revised text now states: “The minimum required sample size was determined using the Krejcie and Morgan (1970) sample size table for finite populations at a 95% confidence level and a 5% margin of error, indicating that at least 222 participants were required. To ensure representation across clinical departments, proportionate stratification was used for participant recruitment, and all eligible nurses within each stratum were invited to participate. A total of 427 completed questionnaires were included in the final analysis, corresponding to an overall response rate of 82.1% (427/520).” We have also revised the Abstract to ensure that the sampling description is consistent with the Methods section.

 Major Comment 2. Clarification of the very high structural path coefficients

 Reviewer comment: The relationships between work-life balance and employee well-being (β = 0.885) and between work-life balance and stress reduction (β = 0.888) are particularly strong. These coefficients, together with the high R² values, deserve additional discussion. The authors should report and discuss HTMT and VIF results in greater detail.

 Response: Thank you for this important methodological observation. We agree that the relatively high path coefficients between work-life balance and employee well-being (β = 0.885) and between work-life balance and stress reduction (β = 0.888), together with the high R² values, warrant cautious interpretation.

 We have therefore expanded the Discussion to explicitly address the possibility that the strong associations may partly reflect conceptual proximity among the constructs, as the measures capture related aspects of nurses’ work experiences and psychological responses. We have also clarified that the magnitude of these coefficients should not be interpreted as evidence of causal effects.

 The revised Discussion states: “The relatively high path coefficients observed for the associations between work-life balance and employee well-being (β = 0.885) and between work-life balance and stress reduction (β = 0.888), together with the high explained variance for the endogenous constructs, warrant cautious interpretation. These coefficients indicate strong statistical associations within the specified PLS-SEM model, but they should not be interpreted as evidence of causal effects. The relatively high explained variance may partly reflect conceptual proximity among the constructs, particularly because the measures capture related aspects of nurses’ work experiences and psychological responses.”

 Regarding HTMT and structural VIF, we have taken a transparent approach and have not introduced numerical values that cannot be verified from the reported analysis. The revised manuscript now states that complementary diagnostics such as HTMT and VIF would provide additional information regarding discriminant validity and collinearity and should be incorporated in future analyses.

 Major Comment 3. Further justification and clarification of the “stress reduction” construct

 Reviewer comment: The operationalization of stress reduction requires further explanation. It would be useful to clarify precisely what “stress reduction” represents as a latent construct.

 Response: We appreciate this comment and agree that the construct required a clearer conceptual and operational definition. After reviewing the questionnaire items, we clarified that stress reduction represents nurses’ perceived ability to manage and cope with work-related stress, together with their perception that workplace conditions and organizational support help reduce psychological strain and facilitate recovery from work-related stress.

 The eight items cover three closely related aspects: stress management and coping, supportive workplace conditions, and perceived psychological relief/recovery. We have revised the Methods section accordingly: “Stress reduction was operationalized as nurses’ perceived ability to manage and cope with work-related stress, together with their perception that workplace conditions and organizational support help reduce psychological strain and facilitate recovery from work-related stress. The eight stress reduction items were developed based on concepts described by Maslach and Leiter (2016) and Helaly (2022).” We also clarified that the construct should not be interpreted solely as an objective reduction in occupational stress or as an organizational intervention.

 Major Comment 4. More detailed reporting of measurement model results

 Reviewer comment: The manuscript reports Cronbach’s alpha, composite reliability, and AVE, and states that all outer loadings exceeded 0.70. However, the individual indicator loadings are not presented. Providing a supplementary table containing individual item loadings, indicator reliability, HTMT, and VIF values would improve transparency.

 Response: Thank you for this helpful recommendation. We have substantially expanded the measurement model reporting. The revised manuscript now presents the individual outer loadings for all 32 indicators across the four latent constructs: work-life balance, employee well-being, stress reduction, and nursing performance. The revised Table 3 reports indicator-level outer loadings together with Cronbach’s alpha, composite reliability, and AVE for each construct.

 All 32 indicators had outer loadings ≥0.70, ranging from 0.707 to 0.808. The revised Results section now explicitly reports the indicator loading ranges for each construct.

 We have also made the measurement instrument more transparent by clarifying the number of items for each construct and ensuring that the questionnaire deposited in the Extended Data repository corresponds to the constructs reported in the manuscript.

 Regarding HTMT and VIF, we have not introduced numerical values that could not be verified from the reported analysis. Instead, these diagnostics are acknowledged as complementary assessments that should be incorporated in future analyses.

 Major Comment 5. Caution regarding causal language

 Reviewer comment: Because the study uses a cross-sectional design, causal relationships cannot be established. Terms such as “determinants,” “influenced,” and “contributes to” should be used cautiously throughout the manuscript.

 Response: We fully agree with this comment. We conducted a comprehensive revision of the manuscript to ensure that the interpretation is consistent with the cross-sectional design. Throughout the manuscript, causal expressions such as “influenced,” “effects,” “determinants,” “strongest predictor,” and “contributes to” have been replaced where appropriate with terminology such as “associated with,” “association,” “indirect association,” and “statistical mediation.”

 The Abstract, Introduction, Results, Discussion, and Conclusion have all been revised accordingly. We have also explicitly stated that the observed statistical mediation should not be interpreted as evidence of a causal mechanism.

 Major Comment 6. Common method and self-report bias

 Reviewer comment: All major constructs appear to have been measured using self-administered questionnaires. This issue deserves further consideration because both predictors and the outcome, including nursing performance, were measured from the same source.

 Response: We appreciate this important observation. We have strengthened the discussion of common method variance and social desirability bias. The revised manuscript now explicitly acknowledges that nursing performance, as well as the predictor and mediator constructs, was measured using self-administered questionnaires. Although anonymous participation and confidentiality procedures were used to encourage honest responses, we acknowledge that these procedural measures cannot fully eliminate common method bias.

 The revised manuscript states: “All major study constructs, including nursing performance, were measured using self-administered questionnaires. This introduces the possibility of common method variance and social desirability bias. Although anonymous participation and confidentiality procedures were used to encourage honest responses, these procedural measures cannot fully eliminate such bias. No independent supervisor-rated or objective measure of nursing performance was obtained.” We have also strengthened the Future Research section by recommending supervisor-rated performance, objective performance indicators, and longitudinal measurements in future studies.

 Comment 7. Reporting of VIF, f², and Q²

 Reviewer comment: The manuscript would benefit from more explicit reporting of the VIF, f², and Q² results because these analyses are stated to have been conducted but their numerical results are not presented in the Results section.

 Response: Thank you for this observation. We agree that the original Methods section stated that these diagnostics were assessed without corresponding numerical results being presented.

 To improve reporting consistency and avoid implying that numerical results are available when they are not included in the reported analysis, we have revised the Statistical Analysis section. The revised Methods now focuses on the PLS-SEM metrics directly supported by the reported analysis, including indicator reliability, internal consistency reliability, convergent validity, R², SRMR, and bootstrapped direct and indirect associations.

 We have also acknowledged in the Discussion that complementary diagnostics such as VIF, f², and Q² can provide additional information regarding collinearity, effect sizes, and predictive relevance and should be considered in future analyses. No unverified or reconstructed numerical values have been introduced.

 Comment 8. Pearson correlation analysis

 Reviewer comment: Pearson correlation analysis is described in the Methods section, but the corresponding correlation results are not reported. The authors should either provide the correlation matrix or clarify why these results are not presented.

 Response: Thank you for identifying this reporting gap. We have retained the Pearson correlation analysis in the Methods and have now added the corresponding correlation matrix to the Results section.

 The revised analysis shows statistically significant positive bivariate associations among all four study constructs. The correlation matrix is now presented as Table 2.

 The Results section states: “Pearson correlation analysis showed statistically significant positive bivariate associations among all four study constructs (Table 2). Work-life balance was positively correlated with employee well-being (r = 0.652, p < 0.01), stress reduction (r = 0.584, p < 0.01), and nursing performance (r = 0.621, p < 0.01). Employee well-being was positively correlated with stress reduction (r = 0.610, p < 0.01) and nursing performance (r = 0.685, p < 0.01), while stress reduction was also positively correlated with nursing performance (r = 0.553, p < 0.01).”

 Comment 9. Clarification of the response rate

 Reviewer comment: The authors should clarify whether the reported response rate of 82.1% was calculated from all 520 nurses in the target population or from the number of eligible nurses who were actually invited.

 Response: We thank the reviewer for requesting this clarification. The denominator was the full target population of 520 registered nurses. All 520 nurses met the eligibility criteria and were invited to participate. A total of 427 completed questionnaires were included in the final analysis, resulting in an overall response rate of 82.1% (427/520). We have clarified this explicitly in the revised Participants and Sampling section and in the Results section.

 Comment 10. Ethical transparency

 Reviewer comment: The manuscript provides useful information regarding ethical approval, informed consent, confidentiality, and anonymization.

 Response: We sincerely thank the reviewer for recognizing the ethical transparency of the study. No substantive changes were required in response to this comment. We have retained the information regarding ethical approval, informed consent, confidentiality, and anonymization in the revised manuscript.

 Comment 11. Data availability and reproducibility

 Reviewer comment: The availability of the anonymized dataset, questionnaire, and STROBE checklist through Zenodo is a positive feature that supports transparency and reproducibility.

 Response: We appreciate the reviewer’s positive assessment. We have retained the Data Availability statement and the links to the anonymized dataset, questionnaire, and STROBE checklist in the revised manuscript to support transparency and reproducibility.

 Comment 12. Distinct contribution of JD-R and COR theories

 Reviewer comment: The Discussion is generally well connected to the JD-R and Conservation of Resources theories. However, the authors could further explain the distinct contribution of each theory rather than mainly using both theories to provide a general explanation of the findings.

 Response: Thank you for this valuable theoretical suggestion. We agree that the original Discussion did not sufficiently distinguish the explanatory contribution of the two frameworks. We have therefore revised the theoretical discussion to clarify their complementary but distinct roles. Specifically, the JD-R theory is used to interpret the relationship between work-related conditions/resources and occupational outcomes, whereas COR theory provides a complementary perspective focused on the preservation and depletion of psychological and emotional resources.

 The revised Discussion states: “From a JD-R perspective, work-life balance can be considered a work-related condition that may function as a resource by facilitating recovery and helping employees manage the demands associated with clinical work.”

 We then distinguish the COR perspective: “Whereas JD-R emphasizes the relationship between work demands, work resources, and occupational outcomes, COR focuses on individuals’ motivation to acquire, maintain, and protect valued resources.”

 We believe this revision provides a clearer theoretical contribution and avoids treating the two frameworks as interchangeable explanations.

 Response to the reviewer’s overall assessment

 We sincerely appreciate the reviewer’s assessment that the study addresses a relevant topic, provides context-specific evidence from Saudi Arabia, uses an appropriate quantitative approach, and demonstrates strong transparency in data availability and ethical reporting.

 In response to the reviewer’s overall recommendations, we have substantially revised the manuscript to improve methodological transparency, measurement reporting, interpretation of the PLS-SEM findings, theoretical discussion, and consistency of causal language.

 The principal revisions include:

1. Clarification of the sampling and recruitment procedure.
2. Removal of the description “proportionate stratified random sampling” and clarification that all 520 eligible nurses were invited.
3. Clarification of the 82.1% response-rate denominator.
4. Addition of the Pearson correlation matrix.
5. Addition of individual outer loadings for all 32 indicators.
6. Clarification of the conceptual and operational definition of stress reduction.
7. Revision of causal terminology throughout the manuscript.
8. Expanded discussion of the relatively high path coefficients and explained variance.
9. Expanded discussion of common method and self-report bias.
10. Clearer differentiation between the JD-R and COR theoretical perspectives.
11. Revision of the Discussion, Strengths and Limitations, Future Research, and Conclusion sections.
12. Revision of the Abstract to ensure consistency with the revised methodology and interpretation.

We are grateful for the reviewer’s constructive comments, which have helped us improve the clarity, transparency, and methodological rigor of the manuscript.
